# The Brain’s First “Traffic Map” through Unified Structural and Functional Connectivity (USFC) Modeling

**DOI:** 10.21203/rs.3.rs-4184305/v1

**Published:** 2024-04-19

**Authors:** Arzu HAS SILEMEK, Haitao Chen, Pascal Sati, Wei Gao

**Affiliations:** Cedars-Sinai Medical Center; Cedars-Sinai Medical Center; Cedars-Sinai Medical Center; Cedars-Sinai Medical Center

## Abstract

The brain’s white matter connections are thought to provide the structural basis for its functional connections between distant brain regions but how our brain selects the best structural routes for effective functional communications remains poorly understood. In this study, we propose a Unified Structural and Functional Connectivity (USFC) model and use an “economical assumption” to create the brain’s first “traffic map” reflecting how frequently each structural connection segment of the brain is used to achieve the global functional communication system. The resulting USFC map highlights regions in the subcortical, default-mode, and salience networks as the most heavily traversed nodes and a midline frontal-caudate-thalamus-posterior cingulate-visual cortex corridor as the backbone of the whole brain connectivity system. Our results further revealed a striking negative association between structural and functional connectivity strengths in routes supporting negative functional connections as well as much higher efficiency metrics in the USFC connectome when compared to structural and functional ones alone. Overall, the proposed USFC model opens up a new window for effective brain connectome modeling and provides a considerable leap forward in brain mapping efforts for a better understanding of the brain’s fundamental communication mechanisms.

## Introduction

As of now, the two main non-invasive imaging approaches for characterizing the brain’s connectome are: structural diffusion-weighted MRI^1^ and resting state functional MRI (rs-fMRI)^2^. Structurally, diffusion-weighted MRI-based tractography approach offers a global view of how distant brain regions are connected through white matter fiber tracts^3^. The human brain's structural connectome is remarkable for its highly organized and modular architecture, facilitating efficient communication and functional specialization^4,5^. Functionally, the resting-state fMRI approach offers a way of measuring “functional connectivity (FC)” by quantifying the degree of blood oxygen level dependent (BOLD) signal fluctuation synchronizations across distant brain regions^6,7^. Based on the “neurons firing together wiring together” principle^8^, FC measures enable the characterization of the human brain functional connectome^9^, which is typically organized into distinct networks including the somatomotor^6^, visual^10^, auditory^11^, default mode network (DMN)^12,13^, salience^14^, and executive control ones^15^. These functional networks are generally believed to directly underlie various primary, cognitive, and socioemotional functions^16,17^. Both the structural and functional connectome feature a small-world network topology, characterized by densely locally interconnected clusters of brain regions and critical long-distance “short cuts” and hubs that bridge inter-cluster communication^18,19^, providing supports for both segregated and integrated information processing, essential for complex cognitive processes^20^.

There are ongoing efforts to unveil the relationships between structural and functional connectomes based on the idea that structural white matter fiber bundles form the foundation for FC or communication^21-28^. Most studies are correlational in nature and their findings support a moderate positive relationship between structural and functional connections (via global modules max (R^2^) ≈ 0.1, via local modules R^2^ ranging between – 0.01 to 0.42)^24,29,30^. However, it is generally accepted that there is not a one-to-one correspondence between these two types of connections since many functional connections exist between brain regions without direct structural connections^31^. Instead, FC could be mediated by multiple segments of structural connections. Given the interconnected nature of the structural connectome, it is likely that there are multiple structural pathways linking these pairs of regions with significant FC without a direct structural link. However, it remains unclear how our brain selects the best structural route for a specific functional connection. New insights into this structural-functional coupling mechanism would shed important light on how our brain works in health and disease.

In this study, we liken the brain to a country with different brain regions being different cities, the brain’s structural connectome corresponding to the road system, and the functional connectome reflecting the amount of people traveling among different cities. Given that there are different routes from one city to another, how people choose their routes will determine the “traffic map” (i.e., the load of each road segment) of the road system. Under this new framework, the goal of this work is to characterize the “traffic map” and reveal the most heavily used structural segments of the brain, which may bear significant implications for better understanding of both normal brain functioning and diseased conditions. To achieve this, we make one important economical assumption that distance and road condition (translating to anatomical distance and structural connectivity (SC) strength in the brain) are the two most important factors for route selection. Based on this principle, we aim to build the brain’s first unified structural and functional connectome (USFC) to uncover its effective “traffic map”. Employing the model, we identified an asymmetric network of brain traffic, characterized by a predominance of pathways originating from the subcortical, default-mode, and salience networks as well as a midline frontal-caudate-thalamus-posterior cingulate-visual cortex corridor that acts as the backbone of the global brain communication system. Our results also accentuate the critical role of stronger structural connections in underpinning significant negative FC, offering fresh perspectives on their functional relevance. Finally, the USFC map exhibits much elevated levels of efficiency, modularity, and betweenness centrality in comparison to conventional structural and FC maps, supporting its superiority in modeling the brain’s superb efficiency in communication. Overall, the USFC model provides a novel framework for modeling the brain's effective connectivity system and potentially opens up a new window uncovering the brain’s working principles.

## 1. Materials and Methods

This study involved 394 subjects from the Human Connectome Project – 1200 Subjects Release (S1200) including behavioral and 3T MRI data. These subjects were randomly selected from the shuffled dataset, constituting one-third of the total sample. We downloaded minimally processed diffusion tensor imaging, T1-MPRAGE and rs-fMRI data to perform structural and FC analysis. Details of the minimal image processing are provided in Glasser et al.^32^.

### 1.1. Structural connectivity

Individual structural networks were constructed through the utilization of whole brain probabilistic fiber tracking with MRtrix3 (www.mrtrix.org) within the subject's space as described in Has Silemek et al.^33^. To generate fractional anisotropy (FA) and mean diffusivity maps, we initially applied diffusion tensor fitting to diffusion tensor imaging data, accounting for head motion and eddy currents, and performed skull stripping procedures using FSL's diffusion toolbox^34^.

To obtain a precise estimation of the fiber orientation distribution (FOD) during constrained spherical deconvolution, we determined the multi-shell, multi-tissue response functions based on FOD values exceeding 0.7 for white matter and lower that 0.2 for gray matter and cerebrospinal fluid^35^. Subsequently, for fiber construction, we employed probabilistic tractography algorithms, which generated a total of 150,000 fibers, with a minimum length threshold set at 20 mm. Default parameters included a step size of 0.2 mm, a minimum radius of curvature of 1 mm, and an FOD cut-off of 0.1. Seeds for tractography were specified using all voxels from 1 mm dilated white matter masks. The tracking of these seeds was confined by the mask’s boundaries and predefined FA or FOD thresholds. Streamlines were mapped onto structural image which was labeled based on the AAL atlas (2009). Following this, we computed the average FA for each fiber after estimating the FA values at each point along the fiber's trajectory as an index of the SC strength for this fiber tract. For each pair of nodes, the mean FA of the fibers that intersect both nodes was calculated, ensuring that the number of fibers in the selected vectors of the nodes matched the number of fibers in the tract structure.

### 1.2. Functional connectivity

The preprocessing steps for FC involved several key procedures, including skull stripping using FSL, segmentation of white matter, gray matter, and cerebral spinal fluid via FSL FAST and motion correction with AFNI (participants with framework displacement > 0.3 mm and < 900 volumes were excluded), bandpass filtering in the frequency range of 0.01 to 0.1 Hz using AFNI, and spatial smoothing via a Gaussian kernel with a full width at half-maximum of 6 mm, non-linear registration of rs-fMRI images to the Montreal Neurological Institute atlas using ANTs. Following preprocessing, global signal regression was applied to remove the mean gray matter signal. Subsequently, FC was computed by measuring the correlation between the average signals of each pair of 90 regions in the AAL atlas (p < 0.05, false-discovery rate (fdr)^36^ corrected).

### 1.3. Unified Structural and Functional Connectome (USFC) Construction

Construction of USFC was performed by a custom MATLAB script including the following procedures:

#### 1.3.1. Template Distance Calculation

First, we defined a standard distance map based on the AAL template extracting the anatomical coordinates for designated brain regions, which were sequentially labeled from 1 to 90. Then, the Euclidean distance between the center of mass of each of the 90 region pairs was determined.

#### 1.3.2. Identifying the most “efficient” pathway

**Figure 1:**
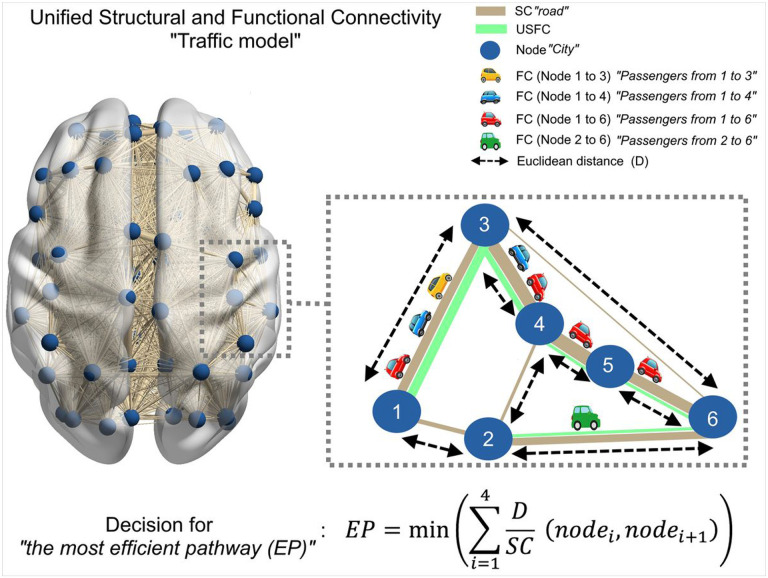
"Traffic model" of unified structural and functional connectivity. The left panel shows a glass brain view representing brain network communication. Our approach, on the right magnified image, unifies structural and functional connectivity to depict brain traffic, likening the brain to a country where each brain region is a city connected by roads (structural connectivity). Passengers (functional connectivity ‘cars’) choose the most efficient route based on road condition (strength of structural connectivity) and distance. Thicker nude-colored edges between nodes (regions) indicate stronger structural connectivity (better road condition). Black dashed arrows represent Euclidean distances between cities. The right panel illustrates four scenarios with different passengers (functional connectivity 'cars'). The red car chooses a path with 4 steps (1-3-4-5-6) due to higher structural connectivity strength, despite a similar distance to a direct connection (3-6). Similarly, the blue car chooses a path with better road condition (1-3-4) over a slightly longer distance (1-2-4). The yellow car opts for a direct connection with moderate road condition (1-3) instead of a longer, unbalanced route (1-2-4-3). The green car selects the shortest direct link with moderate road condition (2-6) rather than a longer route with similar road condition (2-4-5-6). The thickness of the green line represents the sum of passengers on each segment. Thicker green lines indicate routes used by more passengers. Equation for the calculation the most “efficient” pathway is given on the bottom of the panel, where EP is the most efficient pathway, D denotes the Euclidian distance and SC reflects the structural connectivity between each pair of AAL connection. “i” indicates the number of steps (up to 4) that are searched to calculate the least cost.

The cost function was defined as the Euclidean distance of AAL atlas divided by the strength of direct SC between a pair of regions along all potential routes (up to 4 steps were searched). The most “efficient” pathway for each FC in each subject was identified by summing the cost of each “step” and choosing the one with the least “cost” as follows:

(1) $$EP=\text{min}\left(\sum_{i=1}^{4}\frac{D}{SC}(node_{i},node_{i+1})\right)$$

where EP is the most efficient pathway, D denotes the Euclidian distance and SC reflects the structural connectivity between each pair of AAL connection. Schematic demonstration of the most “efficient” pathway is visualized in Fig. 1.

#### 1.3.3. Unified structural and functional connectivity (USFC) value calculation

A USFC value for each “road segment”/direct SC was then calculated as the sum of all FC values that use this segment in their respective routes, essentially quantifying the amount of “traffic” on this “road segment” for each subject (i.e., weighted by both the number and degree of “traffic”) (Fig. 1). After calculating the mean USFC by averaging the values in each pair of connections across the group, one-sample t-test and fdr correction at a threshold p lower than 0.05 were applied.

### 1.4. Structural-functional relationships across all USFC routes:

To better understand the relationships between SC and FC along the defined USFC routes, we performed functional-structural strength correlation analysis at the group level across all routes in four subgroups based the number of steps of the corresponding route, focusing on those that are consistent in over 50% of the subjects. The SC for each step was calculated by averaging the SC values for every pair of nodes within the respective route. Spearman correlation was performed to test the relationship between the SC and FC at each step and p < 0.05 was accepted as significant.

### 1.5. Graph-theoretical metrics:

To examine the information transferring efficiency of the newly derived USFC connectome, we utilized three principal graph-theoretical metrics calculating via Networkx package in Python^37^ to assess weighted network characteristics: efficiency^38-40^, modularity^41^, and betweenness centrality^42^. Efficiency denotes the network's capacity for swift and economical propagation of information. Modularity quantifies the degree to which the network is partitioned into cohesive communities or clusters with dense intra-cluster connections. Betweenness centrality measures the nodes' role in facilitating information flow, thus reflecting their capacity to integrate data across disparate functional regions. These metrics were computed for each individual across various metrics, namely FC, SC, and USFC, and statistical comparisons were made using the t-test.

## 2. Results

### The brain’s first “Traffic Map”

**Figure 2:**
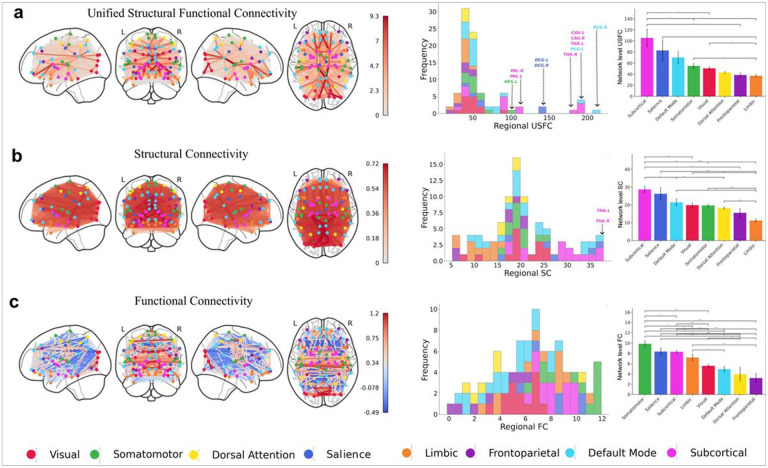
Regional and network characteristics of each connectivity type. Each row in panel illustrates glass brain views of the average 'traffic map' weighted by unified structural-functional connectivity (USFC) (a), structural connectivity (SC) (b), and functional connectivity (FC) (c) matrices from top to bottom, respectively. The histograms in the middle column show the frequency distribution of regional USFC (a), SC (b), and FC (c) values. The x-axis indicates the sum of connectivity values of each node, and the y-axis represents the count of regions within each bin, with bins colored based on the regions' corresponding network involvement. Outliers in USFC (11 regions; HES.L = left Heschl's gyrus, PAL.R and PAL.L = bilateral pallidum, DCG.R and DCG.L = bilateral dorsal cingulate gyrus, THA.R and THA.L = bilateral thalamus, CAU.R and CAU.L = bilateral caudate, PCG.R and PCG.L = bilateral posterior cingulate gyrus) on the histogram (first row of the middle column (a)) are labeled based on the Interquartile Range (IQR) (out of the range between the 25th and 75th percentile), with colors indicating the corresponding network. Likewise, bilateral thalamus is labeled via its network color (magenta) as these were found as outliers in regional SC values (second row of the middle column (b)). Network-level comparisons are presented in the third column for USFC (a), SC (b), and FC (c), with asterisks denoting significant differences between the networks (*: p<0.05, **: p<0.01, ***: p<0.001), and lines indicating standard deviation. Node colors correspond to the respective network as defined by the Yeo Atlas, and bar colors follow the same coding for brain networks. Maps were generated following group-level fdr correction (p<0.05).

The USFC map, characterizing the accumulative “functional load” of each structural connection accounting for the distance (Fig. 1 & Supplementary Fig. 1a), is visualized in the first column of Fig. 2a while the SC and FC maps were presented in the first columns of Fig. 2b, and c, respectively. To better quantify the global distribution of USFC, SC, and FC weights in each brain region, we calculated the overall regional load of each connectivity type and showed their distribution in middle column of Fig. 2. It is immediately clear that USFC featured a long right tail with a set of regions showing much higher values that the rest of the brain (second column of Fig. 2a and Supplementary Table 1). Based on the interquartile range (IQR) calculation^43^, we detected 11 outlier regions (out of the range between the 25th and 75th percentile) with much higher regional USFC values than the rest of the brain, indicating their heaviest involvement in all USFC routes. These regions include the bilateral posterior cingulate gyrus (PCG) in the DMN, thalamus/caudate/pallidum in the subcortical network, dorsolateral cingulate gyrus in the salience network, and left Heschl gyrus [median (IQR): USFC = 48.6 (21.07)] (second column of Fig. 2a & Supplementary Table 1). Two of these outliers (bilateral thalamus) were also highlighted by SC [median (IQR): SC = 19.3 (8.24)] (second column of Fig. 2b), while no outlier was found in FC [median (IQR): FC = 6.69 (4.06)] (second column of Fig. 2c). Consistent with the regional loadings, when examined at network level, the subcortical, the salience and the default-mode network ranked as the top three with highest network-level USFC values (third column of Fig. 2a).

**Figure 3:**
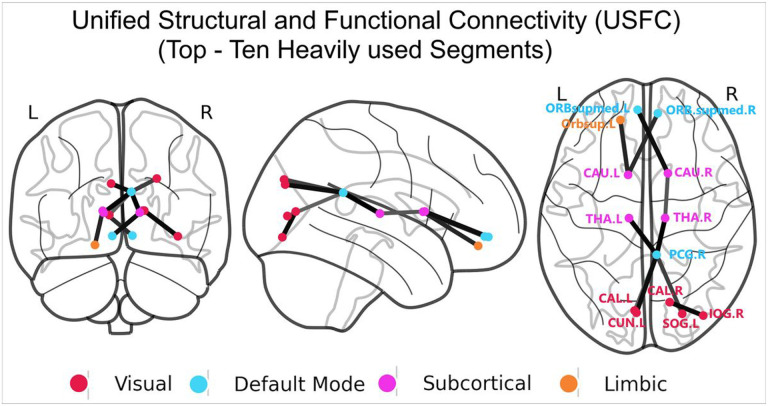
Top ten heavily used segments of an effective “traffic map”. Node colors indicate the relevant network defined by the Yeo Atlas. L = left, R = right, ORBsupmed = orbital part of the superior medial frontal gyrus (blue; default mode network), Orbsup = Orbital part of Superior Frontal Gyrus (orange; limbic network), CAU = caudate (magenta; subcortical), THA = thalamus (magenta; subcortical), PCG = posterior cingulate gyrus (blue: default mode network), CAL = calcarine (red; visual network), CUN = cuneus (red; visual network), IOG = inferior occipital gyrus (red; visual network), SOG = superior occipital gyrus (red; visual network). Edges weighted by USFC values are seen in black.

The ten most heavily used structural pathways based on USFC were shown in Fig. 3. Strikingly, the two hubs of the DMN (i.e., the right PCG and orbital part of the superior medial frontal cortex), were involved in 7 out of the top-10 most heavily used USFC pathways (Fig. 3 & Supplementary Table 2). The bilateral caudate and thalamus were involved in 6 out these top 10 pathways. Together with three connections between the PCG and visual regions (i.e., left calcarine, superior occipital gyrus and cuneus), one connection between the caudate and left superior orbital frontal cortex, and another one between the right calcarine and inferior occipital gyrus, the top 10 most heavily USFC pathways feature a clearly defined, along-the-middle-line, anterior-to-posterior backbone corridor connecting medial frontal to caudate to thalamus and to visual regions (Fig. 3 & Supplementary Table 2).

### Relationships between SC and FC strengths along the defined USFC routes

**Figure 4:**
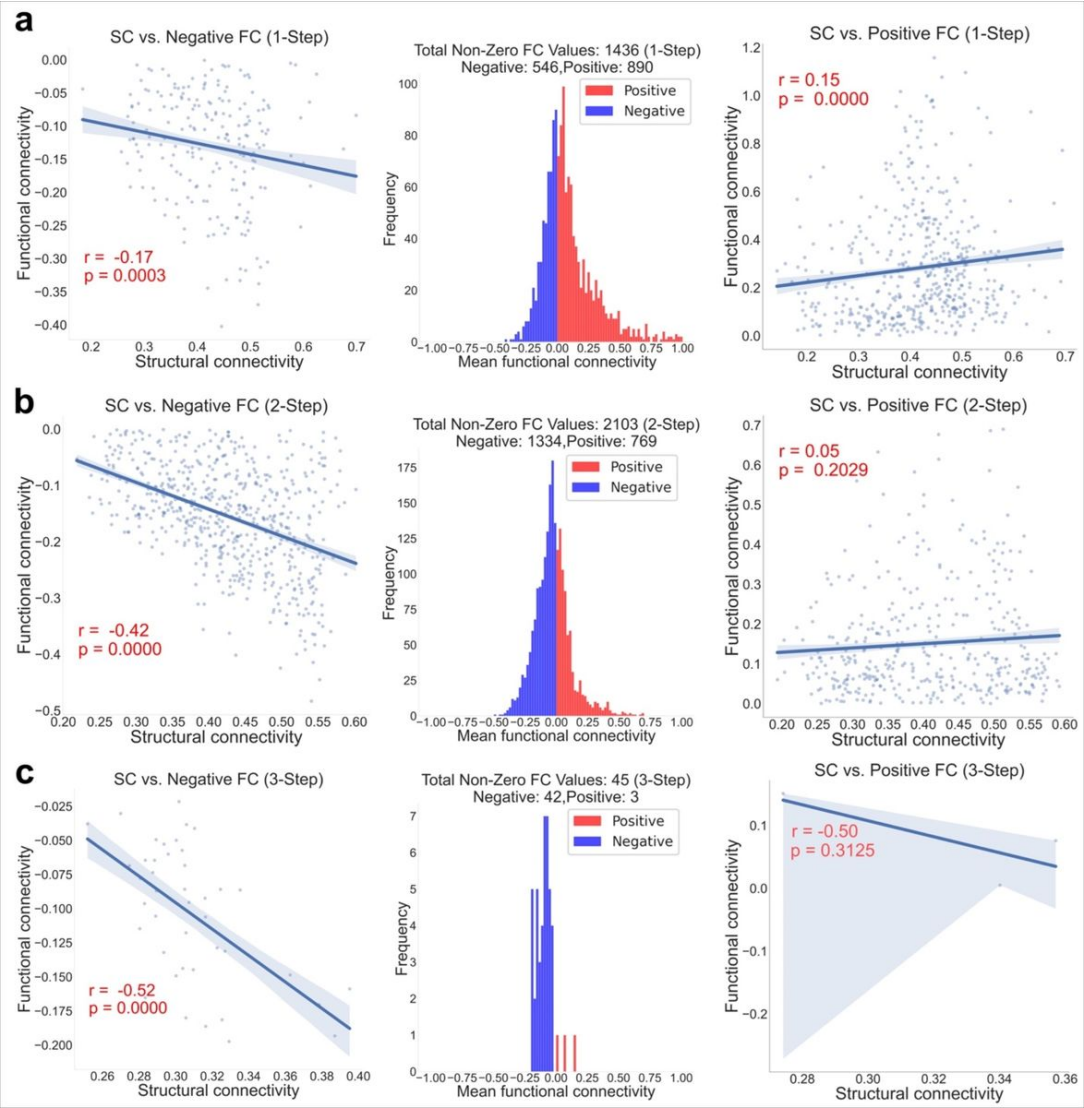
Structural and functional coupling in each step. Each row represents the information about functional and structural connectivity within the steps of USFC routes such as 1-Step (a), 2-Step (b) and 3-Step (c). The scatter plots in first column illustrate the relationships between negative functional connectivity (FC) and structural connectivity (SC) for Step 1 (a), Step 2 (b), and Step 3 (c). Similar demonstrations are provided for the coupling between positive FC and SC for each step (i.e., Step 1 (a), Step 2 (b), and Step 3 (c)) on the third column. Distribution plots in the middle column indicate the number of negative (blue) and positive (red) functional connections in each step, such as Step 1 (a), Step 2 (b), and Step 3 (c).

To better understand the relationships between SC and FC strengths along the defined USFC routes, correlation analysis was done for USFCs at each step for negative (first column of Fig. 4) and positive FCs (third column of Fig. 4) separately. There are 890/769/3 1-/2-/3-step USFCs supporting positive FCs and 546/1334/42 1-/2-/3-step USFCs supporting negative FCs, as shown in the middle column of Fig. 4, with images from top to bottom corresponding to the 1-step, 2-step, and 3-step USFCs, respectively. No common patterns (i.e., shared by > 50% of subjects) emerged for 4-step connections so they were not evaluated. For positive FCs, significantly positive (for 1-step routes) (Fig. 4a, third column) or nonsignificant correlations (for 2 and 3-step routes) (Fig. 4b & Fig. 4c, third column) were observed for routes, which is consistent with previous findings ^24^. Intriguingly, more significant and stronger negative associations were identified for routes underlying negative FCs for all routes raging from 1 to 3 steps (Fig. 4, first column), indicating that stronger negative FC are supported by USFC routes with overall stronger SC.

### Information Transferring Efficiency of USFC:

To examine the information transferring property of the USFC map, three graph-theoretical metrics, namely global efficiency, betweenness centrality, and modularity were calculated and compared between SC, FC, and USFC maps. As shown in Fig. 4, USFC demonstrated superior performances across all three measures, as evidenced by significantly higher global efficiency (p < 0.001) (Fig. 4a), betweenness centrality (p < 0.001) (Fig. 4b) and modularity (p < 0.001) (Fig. 4c). In line with the global measures, significantly superior local efficiency was observed across the entire brain in USFC compared to SC and FC alone (Fig. 4d) (p < 0.001). Higher regional betweenness centrality was observed in regions primarily involved in the DMN, as well as in salience, frontoparietal, dorsal attention, limbic, visual and somatomotor networks (Fig. 4e) (p < 0.001). Higher local modularity was located in salience, frontoparietal, limbic and subcortical networks (Fig. 4f) in USFC compared to FC and SC (p < 0.001).

## 3. Discussion

Based on an economical assumption, our new Unified Structural and Functional Connectivity (USFC) modeling represents the first effort to build a brain’s effective “traffic map” highlighting the brain’s major structural pathways that are most heavily used for efficient functional signal transferring. Based on this model, we revealed a highly skewed brain traffic system featuring the subcortical, the default-mode, and the salience network housing some of the brain's most traversed nodes and a medial frontal-caudate-thalamus-posterior cingulate-visual cortex midline “backbone” corridor as the mostly heavily used structural pathways. Moreover, the finding that stronger structural connections are underlying stronger negative functional connections further supports the functional roles of negative FC and provides a fresh perspective on the dynamic interactions among brain regions. Finally, the significantly higher efficiency, modularity, and betweenness centrality demonstrated in the USFC map when compared with structural and functional connectomes may support the superiority of this “traffic map” in potentially revealing the true working mechanism of the human brain. Overall, the proposed USFC model opens a new window for brain connectome modeling and provides a considerable leap forward in brain mapping efforts by offering a more intricate depiction of the brain’s connectivity landscape.

### The heavily skewed “traffic map” that features the central role of the DMN in USFC.

Our analysis uncovered an striking pattern within the brain's USFC blueprint: the DMN regions collectively possess the third highest nodal USFC values while more strikingly, seven of the top ten most heavily trafficked pathways involve either the PCG or medial prefrontal cortex, the two hub regions of the DMN^44^. Centrally located and occupy a large portion of the brain, the DMN is known for being “active” during rest and its versatile roles in self-reference, social cognition, episodic and autobiographical memory, language, sematic memory, among others^45-47^. All these functions involve complex communications within and between DMN and other brain regions which likely underlies our finding of its central role in the newly defined USFC system. Specifically, the prominent inter-network connections between the DMN hubs and subcortical/visual regions as shown in the top ten USFC pathways likely underscore the DMN’s potential integrative role across different domains, which is highly in line with findings demonstrating DMN’s active and dynamic reorganization of its connectivity patterns across a range of cognitive and socioemotional tasks^48-51^. This finding provides another critical piece of evidence from a global brain “traffic map” perspective that the DMN's role likely goes beyond a passive default state but rather globally contributes to the brain’s efficient signal processing across task domains^49,50^. Overall, our finding of the central role of the DMN in the newly defined USFC system provides new support/explanation for its established importance in development^50,52^, normal adult functioning^48-51,53-55^, aging^56,57^ and various brain disorders^58-61^.

### The importance of subcortical/salience networks in USFC and midline “backbone” corridor

Beyond DMN connections, six of the top-ten most heavily trafficked segments involve the thalamus/caudate while at a network level, the subcortical and salience network regions collectively rank as the two mostly traversed networks in the whole brain “traffic map” ranking (Fig. 2). Regarding the salience network, although not highlighted in the top ten mostly heavily used pathways, its regions collectively rank second in the whole brain traffic map system and the middle cingulate cortex was detected as one of the “outliers” with the highest USFC loadings. These findings are consistent with its reported role of lying on the apex of the brain’s global coordination system by performing a “switching” role among large scale functional networks, especially between the DMN and dorsal attention networks^48,51,62,63^.

The subcortical regions, in particular the thalamus's prominence in this traffic system is consistent with not only its known role as an “relay center” connecting peripheral neural system with the brain cortices but also its versatile involvement in modulating and refining sensory data, shaping consciousness, and enhancing cognitive functions^64-66^. Its highly utilized connectivity with the PCG may be particularly indicative of a sophisticated mechanism that merges external sensory inputs with internal states, an essential process for coherent cognitive function^67^. Similarly, the caudate nucleus not only plays a critical role in movement planning and execution but also serves in a multitude of essential brain functions, including learning, memory, reward, motivation, emotional regulation, and aspects of romantic interaction^68,69^. Structurally, frontal regions are known to be connected to the caudate, which in turn is connected to the thalamus, and subsequently projecting to PCG, providing SC support for the observed medial frontal-caudate-thalamus-posterior cingulate -visual pathway that leads the most heavily USFC segments. The finding of a clearly defined midline corridor connecting frontal to caudate to thalamus to posterior cingulate and finally to visual cortices supporting the most “traffic” in the brain through USFC modeling is striking and opens up new windows for better understanding of the “backbone” structure of the brain’s global communication system. Consistent with our findings, Hagman et al have previously delineated the SC hubs of the human brain and similarly detected a midline “structural core” linking precuneus to posterior, middle, anterior cingulate cortex and finally to medial orbital frontal cortices^4^. However, their examinations exclude subcortical areas so the potential “bridging”/ “disseminating” (e.g., the thalamus) role of subcortical regions were not counted for. With combined consideration of both functional and SC and including both cortical and subcortical regions, the midline corridor delineated in this study featuring frontal-subcortical-parietal-occipital links may have better captured the “backbone” of the brain’s global communication system and deserves more attention in future search of its relevance in health and disease.

### The intriguing finding of strong structural underpinnings of negative FCs.

The finding of moderate but significant positive correlations between SC and FC strengths associated with positive FCs is in line with previous reports^24,70^. However, the finding that routes underpinning negative FCs show a robust negative relationship between SC and FC strengths across one-to-three step connections is more intriguing. Ongoing debate regarding global signal regression and the consequent observation of negative correlations (anti-correlations), underscores the lack of consensus on a singular method for processing resting state data to uncover the 'true' nature of brain functionality^71^. Contrary to the notion of negative FC as a mere byproduct of signal processing, emerging research posits it as a salient aspect of the brain's functional architecture defining modularity of the resting-state fMRI connectome, deeply linked with its structural framework^12,72-77^. Our findings add to the evidence supporting the functional significance of negative FCs after global signal regression and suggest that the brain utilizes a delicate traffic system to choose the best routes (i.e., composed of segments with stronger SC) for negative interactions across different brain regions. Notably, Skudlarski et al. indicated that regions with negative functional FC are not necessarily disconnected structurally^78^. Instead, there is an implication of a complex relationship where structurally close regions can exhibit negative FC, suggesting an intricate coordination of brain dynamics. However, we have to point out that the “one-step” route delineated in this study should not be confused with “direct SC” or “connected by a single white matter bundle” give the limitation of diffusion-weighted imaging-based tractography. In other words, the one-step SC used in this study was derived based on probabilistic tractography and as long as there is a “connected structural route” connecting two brain regions, we define these two regions are “structurally connected” and treat them as “one-step” connections. It is possible that multiple white matter fiber bundles are underlying each of these “one-step” structural connection and the accumulated phase lag across the multiple structural connections may have contributed to the observed negative FC^79^. Compared with the relationships associated with negative FCs, where all three step groups (i.e., 1–3) show significant negative correlations, the relationships associated with positive FCs only show positive relationships for 1-step route. One potential explanation could be that choices for multiple-step positive FCs are more abundant than those for negative FCs and SC is not necessarily a limiting factor, and the choices are not as tightly regulated, resulting in weaker SC-FC correlations. Regardless, the finding that stronger structural routes are underlying stronger negative FCs provides further support for the importance of negative FCs in the brain's efficient/effective communication and functioning.

### The USFC-based connectome demonstrates significantly higher communication performance than both the FC and SC systems.

For all three measures of the brain system communication effectiveness, namely global efficiency, modularity, and betweenness centrality, the USFC-based connectome demonstrates significantly higher performance than both the FC and SC systems. These findings support the potential superiority of the USFC system in depicting the brain’s signal transferring efficiency. Essentially, only looking at the “road system” (i.e., equivalent to the brain’s SC system) or the final “number of people traveling between any two cities” (i.e., equivalent to the brain’s FC system) could not provide a clear picture of the brain’s “traffic patterns” while it is this traffic pattern that directly unveils how the road system effectively work to support the between-city travelling (i.e., signal transferring). The much higher global efficiency and betweenness centrality is likely supported by the highlighted most heavily utilized routes between major functional works while the higher modularity may result from the more densely connected local systems within USFC.

Although this work provides a new perspective on brain connectome modeling, there are several major limitations associated with the current version of USFC that deserve future improvements. First, we made the economic assumption (i.e., shorter distance and stronger SC) for route selection but the “real-time traffic” is not considered in this formula. In other words, future improvement could further consider the current “traffic” along each route (i.e., real-time modeling of the “dynamic” FC^80^) in determining the optimal route between two brain regions. Second, as mentioned above, direct structural connection in this study might not represent one single fiber bundle the 1-step routes may consist of multiple white matter fiber bundles, which bears critical implications on the understanding of SC-FC relationships, particular those with the negative FCs. Finally, we used average FA along the tracts to index SC strength but there are other metrics too (e.g., number of fibers) worth further consideration.

Overall, the USFC model presents a compelling new framework to model the brains “effective connectome” and opens a new window for future research aimed at deciphering the enigmatic principles that govern the brain's efficient communication system. By highlighting the “most-heavily-used brain pathways/networks” in its current version and pursuing continued efforts to refine/navigate this complex "traffic" in both normal and diseased populations, the implications from this new model may reach far into the realms of neuroscience, with the potential to transform both theoretical models and clinical/intervention approaches.

## Figures and Tables

**Figure 5:**
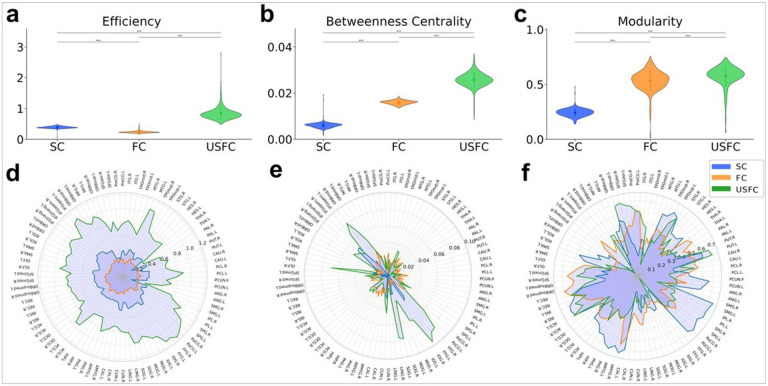
Comparative analysis of graph-theoretical metrics across connectivity types. Violin plots depict the distribution of global efficiency (a), betweenness centrality (b) and modularity (c) for unified structural-functional connectivity (USFC, Green), functional connectivity (FC, Orange), and structural connectivity (SC, Blue). Asterisk (***) corresponds to a significant difference between connectivity types (t-test, p < 0.001). Dots indicate the mean of a specific graph metric for each connectivity type. Lines represent the standard deviation. Radar plot representing the nodal efficiency (d), betweenness centrality (e) and modularity (f) across 90 brain regions for three different connectivity types: SC, FC, and USFC. Each axis of the radar plot corresponds to a distinct brain region, and the distance from the center to a point on a line represents the graph-theoretical metric value for that region. The SC (blue), FC (orange), and USFC (green) connectivity types are depicted as separate lines, allowing for a direct comparison of nodal efficiency across different types of connectivity within each brain region.
